# Concentration gradient induced morphology evolution of silica nanostructure growth on photoresist-derived carbon micropatterns

**DOI:** 10.1186/1556-276X-7-496

**Published:** 2012-09-03

**Authors:** Dan Liu, Tielin Shi, Shuang Xi, Wuxing Lai, Shiyuan Liu, Xiaoping Li, Zirong Tang

**Affiliations:** 1 Wuhan National Laboratory for Optoelectronics, Huazhong University of Science and Technology, Wuhan, 430074, China; 2 State Key Laboratory of Digital Manufacturing Equipment and Technology, Huazhong University of Science and Technology, Wuhan, 430074, China

**Keywords:** Silica nanostructure, Morphology, Concentration gradient, Evolution, Micropattern, 62.23.St complex nanostructures, 61.46.Np structure of nanotubes, 85.40.Hp lithography, masks and pattern transfer

## Abstract

The evolution of silica nanostructure morphology induced by local Si vapor source concentration gradient has been investigated by a smart design of experiments. Silica nanostructure or their assemblies with different morphologies are obtained on photoresist-derived three-dimensional carbon microelectrode array. At a temperature of 1,000°C, rope-, feather-, and octopus-like nanowire assemblies can be obtained along with the Si vapor source concentration gradient flow. While at 950°C, stringlike assemblies, bamboo-like nanostructures with large joints, and hollow structures with smaller sizes can be obtained along with the Si vapor source concentration gradient flow. Both vapor–liquid-solid and vapor-quasiliquid-solid growth mechanisms have been applied to explain the diverse morphologies involving branching, connecting, and batch growth behaviors. The present approach offers a potential method for precise design and controlled synthesis of nanostructures with different features.

## Background

In recent years, nanomaterials with diverse configurations have been investigated actively to explore their different properties which are strongly dependent on the internal and external structural features [1-3]. Various silica nanostructures and their assemblies have demonstrated unique physical, chemical, and optical properties [4-7], which can be used for a wide range of applications. For example, silica nanoparticles with solid or porous structures have been applied for biomedical imaging or theranostic applications as nanoprobes after introducing new functional groups [8]. Silica nanotubes with tubular hollow structures would not only realize easier surface functionalizations on both the outer and inner walls, but would also show potential applications in bioanalysis [9], biocatalysis, [10] and optical devices [11]. Rope-like silica nanowire assemblies showing reversible blue light emission behavior in photoluminescence and infrared analysis could be used to fabricate effective optoelectronic devices and optical signal humid sensors [12]. Theoretical calculations have also been conducted to explore some interesting optical properties of silica nanoclusters [13,14]. Up to the present, many approaches have been introduced to synthesize silica nanostructures [15-19], and various morphologies were reported, such as nanowire assemblies [17,20], nanoflowers [21], nanotubes [22], and nanosprings, [23] etc. The vapor–liquid-solid (VLS) mechanism has been widely applied to explain the growth process above-mentioned [24]. The critical process parameters such as temperature and the growing sources of Si and O elements are always considered to be important factors affecting the synthesis of silica nanostructures. Temperature-dependent growth of silica nanostructures with different morphologies has been reported [17,20]. The growth of silica nanowires under the effect of oxygen-to-Ar carrier gas ratio has been investigated [25]. However, the growth dependence of Si vapor source concentration is seldom reported.

Recently, we reported that the Si vapor source can be generated through high temperature annealing of silicon substrate coated with a thin copper layer in a quartz tube furnace [26]. Different from our previous work, where a method was introduced to selectively grow silica nanowires on the photoresist-derived three-dimensional (3D) carbon posts at the annealing of high temperature, we mainly investigate the effects of local Si vapor source concentration gradient on the growth behavior of silica nanostructures in the present study. It has also been shown in our previous work that copper is a good candidate to catalyze the growth of silica nanowires on photoresist-derived carbon microstructures; therefore, Cu is further to be applied as the catalyst in the present work. Through the design of experiments, silica nanostructures with diverse morphologies of evolution induced by the concentration gradient are obtained, and the growth mechanisms such as VLS and vapor-quasiliquid-solid (VQS) are discussed. The study is meaningful in both the development of controllable synthesis of nanostructures and the integration of nanostructures on microstructures for a variety of electrochemical and bio-nanotechnology applications [27].

## Methods

The typical process flow of our method has been reported earlier, involving main processes of photolithographic patterning of negative photoresist on silicon substrate, magnetron sputtering of thin copper layer, and thermal heating [26]. An SU-8 photoresist (MicroChem Nano SU-8 100, MicroChem Corp, Newton, MA, USA) was applied in this work with phosphorus-doped n-type silicon <100 > wafer as the substrate. Ultrahigh-purity N_2_and H_2_were used as carrier gases during the thermal heating process to carbonize the patterned photoresist.

Firstly, thick photoresist was spin-coated on a cleaned Si/SiO_2_ (5-nm native oxide layer) wafer. Secondly, a soft bake process at 40°C for 30 min and 120°C for 2 min was carried out, followed by exposure performed by a Karl Suss MJB3 contact aligner (SUSS MicroTec, Garching, Germany) for 80 s at 150 mW/cm^2^. A post bake process was then carried out at 95°C for 30 min. Following a 1-h delay time, development was carried out using an SU-8 developer from MicroChem to obtain a patterned 3D photoresist sample. A Cu layer of about 30 nm was then sputtered on the sample, followed by a carbonization process in an alumina tube furnace. Before heating, the furnace was evacuated to 10^−3^ Torr; N_2_ gas flow was introduced (2,000 sccm). In the carbonization process, the furnace was firstly heated to 300°C at the rate of 15°C/min for 40 min with the introduced 2,000 sccm of N_2_ gas flow as the atmosphere; then the sample was heated up to the growth temperature at the rate of about 15°C/min in the atmosphere of 150 sccm of H_2_ gas flow and maintained at the growth temperature for 120 min with the atmosphere changed to 2,000 sccm forming gas (5% H_2_ in N_2_). After the carbonization process, the heater was turned off, and the samples were naturally cooled down in N_2_ atmosphere to room temperature. The temperature was set as 950°C and 1,000°C, respectively, to investigate the growth of silica nanostructures. Special configurations were designed to investigate the effect of Si vapor concentration variations on the silica nanostructure growth. In the configuration, the downstream part of each sample was with patterned SU-8 photoresist micro-post array and sputtered Cu layer, while the upstream part was Si substrate-coated with only a thin layer of Cu, which was designed to generate local concentration gradient at a microscale of Si vapor source for the downstream part. It has been shown that Si vapor source could be generated from thin Cu layer-coated Si substrate at the annealing of high temperature [26]; therefore, the Si vapor source could be controlled by the size of bare Cu-coated Si substrate, which would flow gradually down to the region of 3D carbon-post-patterned Si substrate with the flow of carrier gas. Since the growth of silica nanostructures on 3D carbon posts will consume the Si vapor, Si vapor concentration gradient will be generated along the flow from the upstream to the downstream. It is obvious that the area of the Cu-coated Si substrate, the distance from the upstream to the downstream, and the density of the 3D carbon posts will affect the local Si vapor concentration gradient. Moreover, the overall Si vapor source concentration could also be controlled by the annealing temperature since the generation process of Si vapor source is influenced greatly by the temperature. In general, the upstream is with a higher concentration of Si vapor, while the downstream is with a lower one due to the consumption. During the experiment, the whole length of the sample was about 10 mm along the gas flow, while the length of the 3D post-patterned region was about 7 mm.

The morphologies of the obtained products were investigated by scanning electron microscopy (SEM) (Quanta 200, FEI Company, Hillsboro, OR, USA) equipped with an energy-dispersive X-ray (EDX), and the nanowires were further characterized and analyzed by transmission electron microscope (TEM) and high-resolution TEM (HRTEM) (Tecnai 12,Philips Tecnai, Amsterdam, The Netherlands) equipped with an EDX.

## Results and discussion

### Concentration gradient induced results at a temperature of 1,000°C

**Figure 1:**
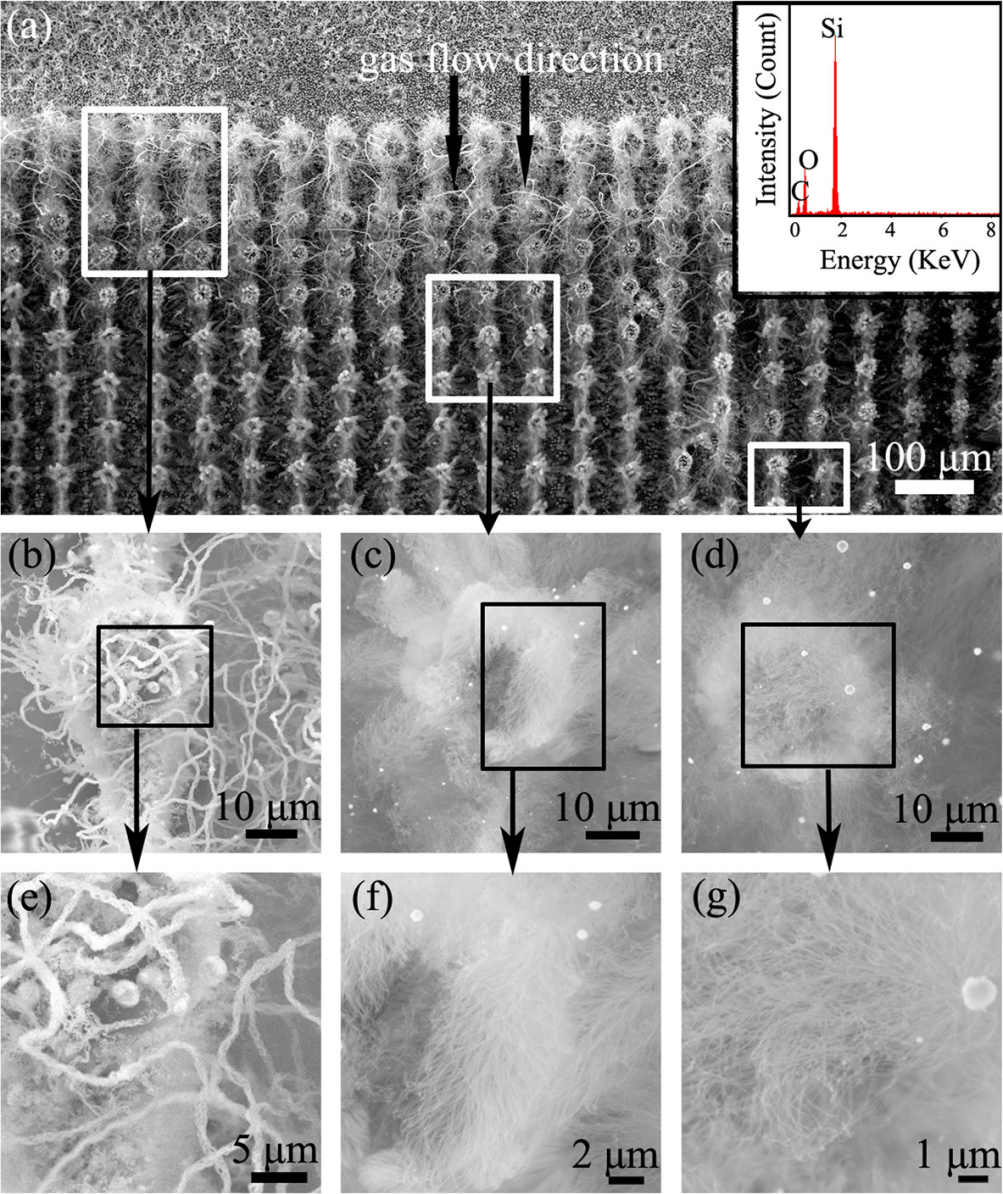
**SEM images of evolved nanostructures grown at the growth temperature of 1,000°C.** (**a**) Overall top-view SEM image of the sample where the gas flow direction is indicated by the double arrows. Insert is the EDX analysis of the nanostructures. (**b**) SEM image of a single post from the upper part indicated in (**a**). (**c**) SEM image of a single post from the middle part indicated in (**a**). (**d**) SEM image of a single post from the bottom part indicated in (**a**). (**e**, **f**, **g**) Enlarged SEM images of (**b**), (**c**), and (**d**), respectively.

Figure 1 shows typical SEM images of the results obtained at a temperature of 1,000°C. The typical overall view of the obtained sample is shown in Figure 1a, where the integrated nanostructures are obviously observed on carbon posts at the downstream part, and the nanostructure density decreased gradually along the carrier gas flow direction. In general, the growth area can be divided into three parts for three different types of morphologies with evolution along with the concentration gradient flow. SEM images of the representative structures from each part are presented in Figure 1b,c,d, and their enlarged SEM images are presented in Figure 1e,f,g, respectively. In Figure 1e, rope-like nanowire curved assemblies with length up to hundreds of micrometers are observed on the carbon post. In Figure 1f, feather-like nanowire assemblies with abundant branches are observed, while octopus-like nanowire assemblies with abundant branches are observed in Figure 1f. Cu catalyst particles are always found at the tip of the assemblies, which could be explained by VLS mechanism [26]. It shows that Si vapor source concentration gradient at microscale has great effect on the nanowire growth rate and the morphology evolution. With the Si vapor flowing from the upstream to the downstream at the designed sample, rope-like structures are grown at the region with the highest Si vapor source concentration, octopus-like ones are grown at the region with the lowest Si vapor source concentration, and feather-like ones are observed at the middle region. Higher Si vapor source concentration leads to a much higher growth rate and a larger amount of nanostructures, and the gradual decrease of Si vapor source concentration could lead to the evolved change of the morphology for grown nanostructures.

**Figure 2:**
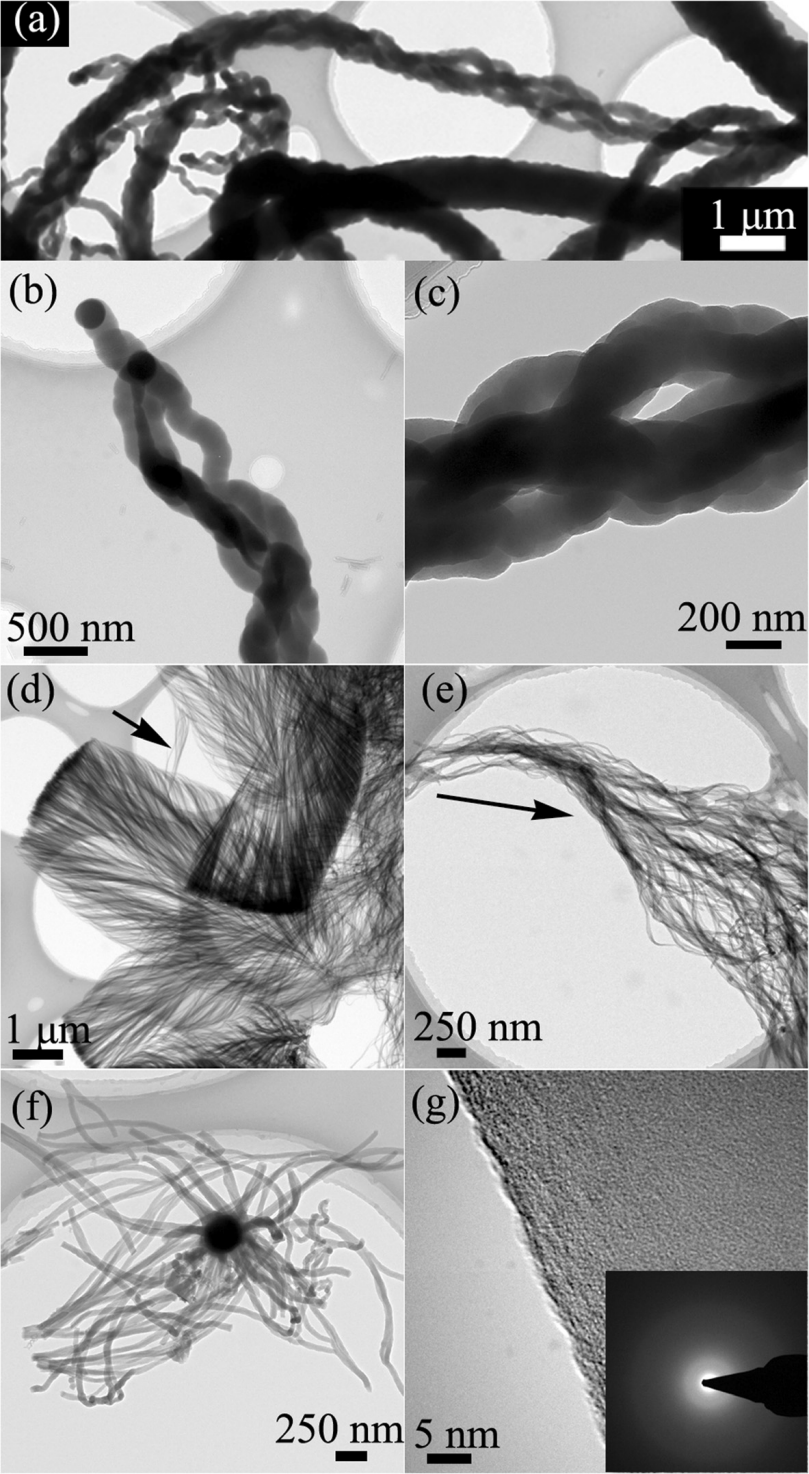
**TEM images of silica nanostructures obtained at 1,000°C.** (**a**) TEM image of part of ultra-long rope-like nanowire assemblies. (**b**) TEM image of rope-like nanowire assemblies with the catalyst at the tip. (**c**) TEM image of enlarged stem view of the rope-like nanowire assemblies. (**d**) TEM image of several feather-like nanowire assemblies. (**e**) TEM image of a fragment of the feather-like nanowire assemblies with the growth direction indicated by the arrow. (**f**) TEM image of the octopus-like nanowire assemblies. (**g**) HRTEM image of the nanowire morphology with the corresponding SAED as an inset.

TEM images of the nanowire assemblies were shown in Figure 2. Figure 2a shows part of the typical ultra-long rope-like nanowire assemblies with different thicknesses. As shown in Figure 2b, nanowire branching behavior could be observed close to the catalyst tip, where a nanowire with a diameter of 200 nm was split into two branches with diameters of 150 and 300 nm, respectively, during the growth process. Multi-branching and connecting led to thicker ropelike assemblies as shown in Figure 2c. The branching behavior could be explained by VQS mechanism, in which catalyst particles in the presence of Si (silica) may undergo melting even at a temperature lower than the metal/Si eutectic temperature and can thus serve as droplets for the growth of the nanostructure branches [28,29]. A typical TEM image of feather-like nanowire assemblies is shown in Figure 2d, showing the length of about 7 μm and width of about 4 μm. It is also revealed that the feather-like structure has symmetrical features, and a small broken part of the assemblies indicated with arrow shows branching or splitting behaviors of the nanowire assemblies. The branching behavior is further confirmed by the TEM image of a large fragment of the feather-like nanowire assemblies shown in Figure 2e, where the great increase on the amount and volume of the nanowires along the growth direction is due to splitting growth [30]. A typical TEM image of octopus-like nanowire assemblies is shown in Figure 2f, implying that many nanowires grew simultaneously on one catalyst particle while several branching behaviors are also observed. The similar phenomenon of nanowires growing from one catalyst was also reported by KM Sunkara and associates as early as 2001 [31], and it could rely on the cause that the fragmentation of one catalyst particle makes it become many smaller catalyst particles under the influence of thermodynamic imbalance and surface energy [32]. Figure 2g shows the typical HRTEM image of one nanowire without any crystalline domains, indicating that the nanowire is amorphous, which is confirmed by the selected area electron diffraction (SAED) pattern with only diffusive rings instead of diffraction spots, shown as an inset in Figure 2g. Optical devices will have more functions on each chip if silica nanostructures with different morphologies originating from silica nanowires that are integrated [11,13,14].

### Concentration gradient induced results at a temperature of 950°C

**Figure 3:**
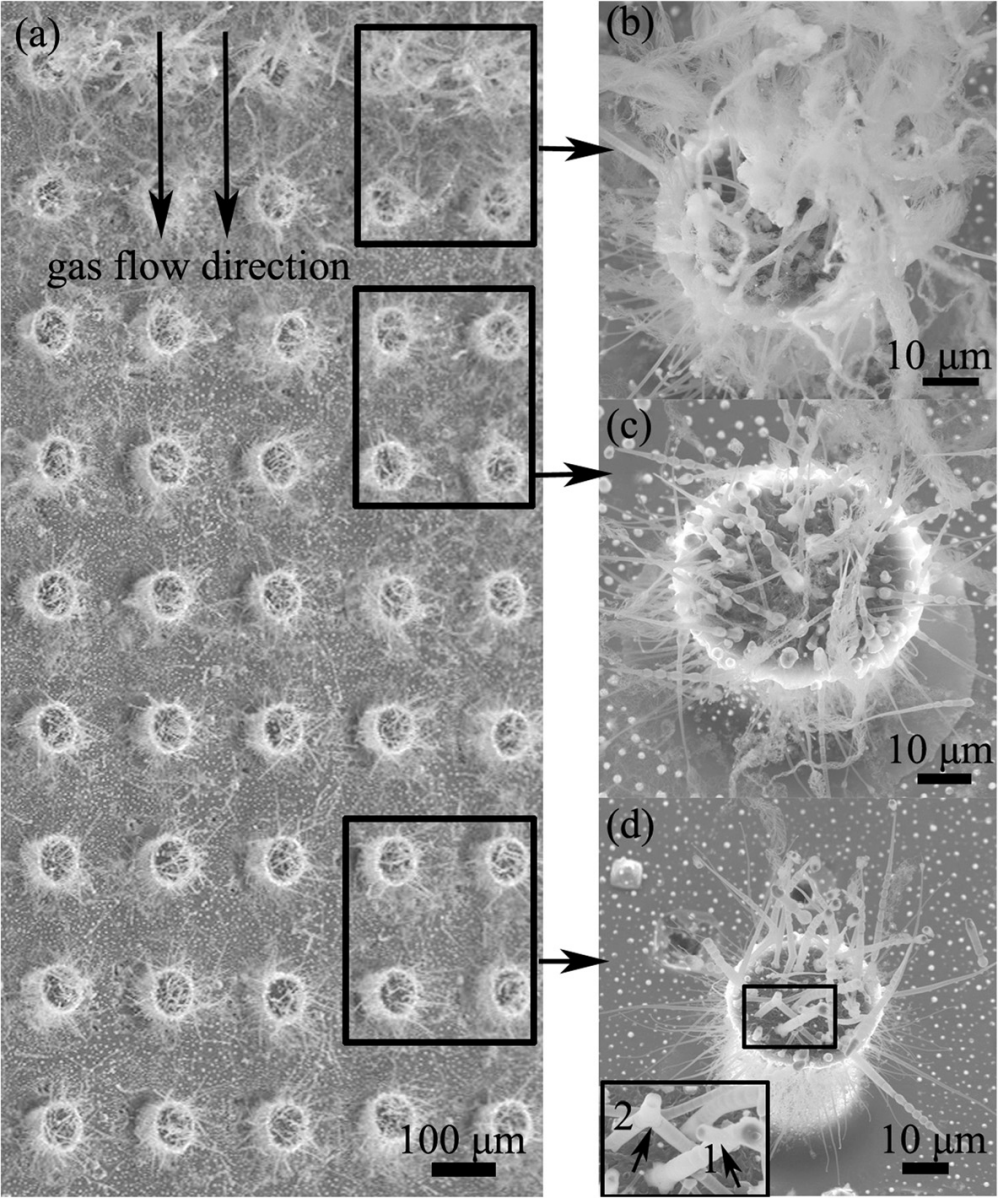
**SEM images of evolved nanostructures grown at 950°C.** (**a**) Overall top view SEM image of the sample obtained with the concentration gradient of Si vapor source, where the gas flow direction is indicated by double arrows. (**b**) SEM image of a single post from the upper part indicated in (**a**). (**c**) SEM image of a single post from middle part indicated in (**a**). (**d**) SEM image of a single post from the bottom part indicated in (**a**).

Figure 3 shows typical SEM images of nanostructure-integrated carbon posts obtained at the temperature of 950°C with concentration gradient of Si vapor source. The overall view of the sample is shown in Figure 3a, where the integrated nanostructures are obviously observed and the nanostructure density decreased gradually along the carrier gas flow direction. In general, the growth area can be also divided into three parts. SEM images of the representative structures from each part are presented in Figure 3b,c,d, respectively. In Figure 3b, stringlike nanowire assemblies with the length up to hundreds of micrometers are observed. In Figure 3c, bamboo-like structures are observed while a few stringlike nanowire assemblies could also be observed. In Figure 3d, only bamboo-like structures are observed. In general, the concentration gradient of the Si vapor source also affects the formation of nanostructure morphology to cause a slight evolution at 950°C, and the dominant mechanism still follows the VLS.

**Figure 4:**
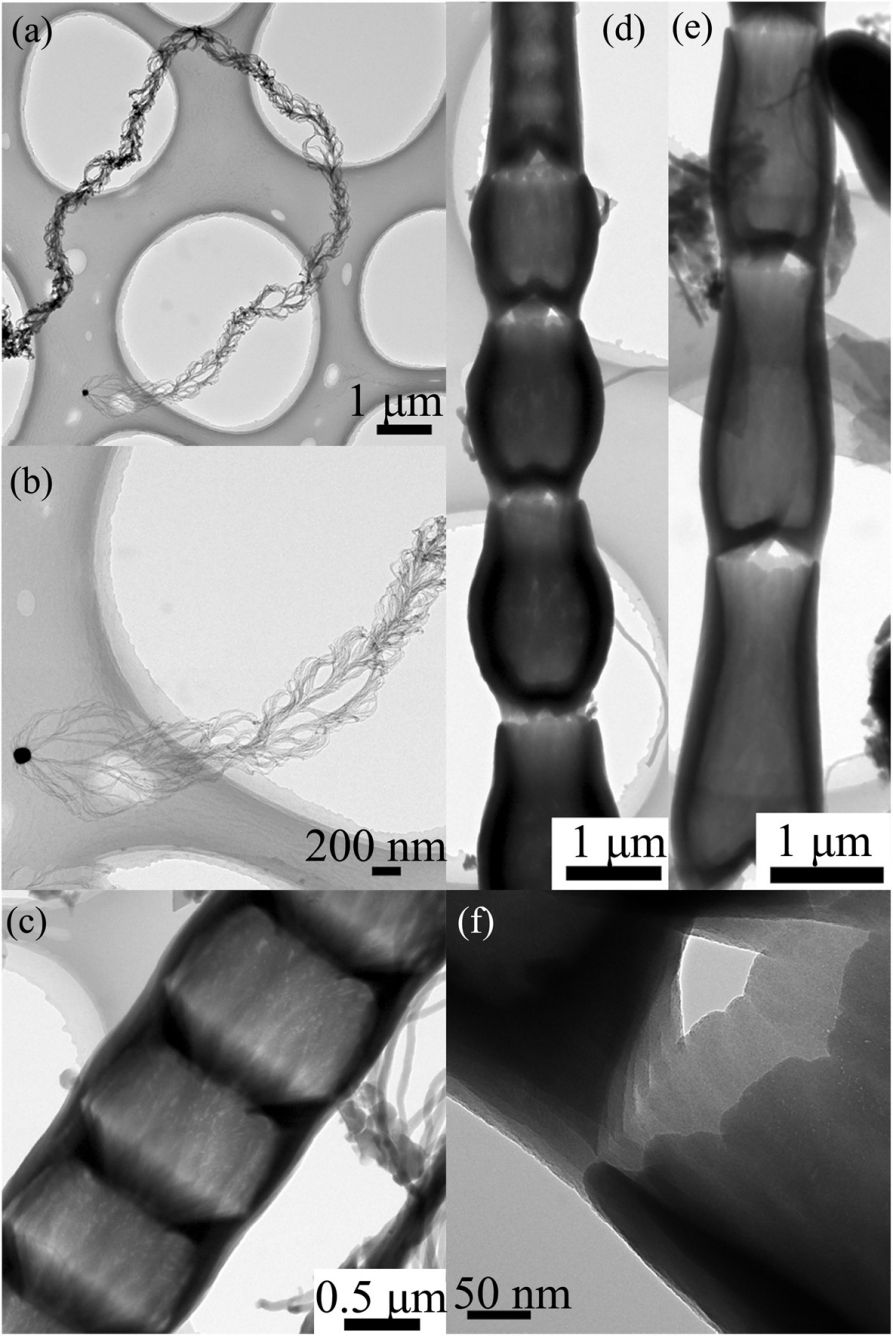
**Typical TEM images of nanostructures obtained at 950°C at the upper part of the sample.** (**a**) TEM image of stringlike nanowire assemblies. (**b**) TEM image of stringlike nanowire assemblies with the catalyst at the tip. (**c,d,e**) TEM images of a bamboo-like nanostructure with large bowl-like, cuplike, and vase-like joints, respectively. (**f**) HRTEM image of the gap between the two joints.

Unique TEM images of the nanostructure and assemblies from Figure 3b,c regions were shown in Figure 4. Figure 4a shows the typical morphology of a stringlike nanowire assembly with curly features. Figure 4b shows the enlarged view of the head part of the assembly with catalyst tip, indicating that many tiny nanowires grew from the catalyst particle with branching and connecting behaviors [12]. Typical bamboo-like structures with large bowl-like, cuplike, and vase-like joints are observed and shown in Figure 4c,d,e, respectively, which were seldom observed from the Figure 3d region. In Figure 4c, a bamboo-like structure is with bowl-like joints having an outer wall diameter of about 400 nm and a wall thickness of about 50 nm. In Figure 4d,e, the length and diameter of the joint are at a microscale. HRTEM image of the gap between the microscale joints is shown in Figure 4f, and it shows the obvious hollow feature with the outer wall composed of several layers, and the inner wall could be composed of aligned nanowires [20]. The gabs further indicate the batch growth for the bamboo-like structures [30]. Furthermore, all the large joints and the gabs implied that the higher concentration has a great impact on the diverse morphologies of the silica structures to cause the evolution behavior.

**Figure 5:**
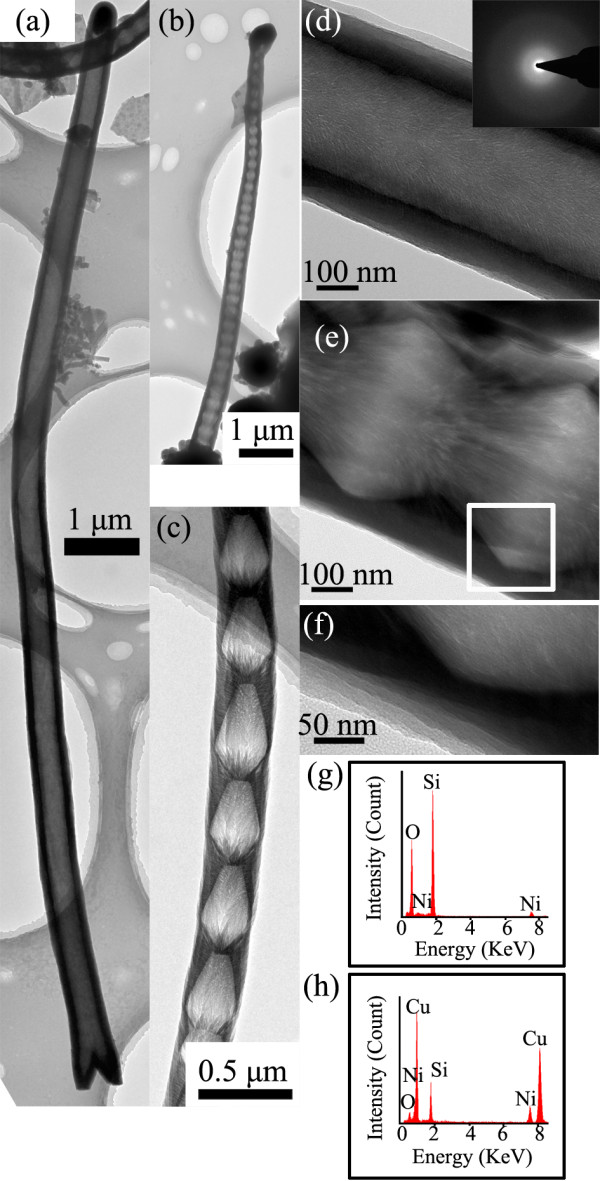
**TEM characterization of nanostructures commonly obtained at the growth temperature of 950°C.** (**a**) TEM image of a tubelike nanostructure. (**b**) TEM image of a bamboo-like nanostructure with bowl-like joints. (**c**) TEM image of a bamboo-like nanowire with dumbell-like joints. (**d**) HRTEM image of tubelike nanostructure with the SAED as an inset. (**e**) HRTEM image of nanostructures with dumbell-like joints. (**f**) Enlarged view of the part marked with a white rectangle in (**e**). (**g**) Typical EDX analysis of nanowires. (**h**) Typical EDX analysis of a catalyst tip.

Silica nanostructures grown at the temperature of 950°C are shown more in Figure 5, where three types of hollow structures are observed as shown in Figure 5a,b,c, respectively. As shown in Figure 5a, the tubelike structure has an outer diameter of about 450 nm, a length up to 15 μm, and a wall thickness of about 50 nm. A bamboo-like structure with bowl-like joints is shown in Figure 3b, having an outer diameter of about 400 nm, a length up to 10 μm, and a thickness of about 50 nm. Both of their outer and inner diameters increase continuously from the catalyst tip to the larger end, which causes the wire to have a uniform wall thickness. Another type of bamboo-like structure with dumbbell-like joints is shown in Figure 5c, having an uneven wall thickness along the wire. It is distinctively observed that for bowl-like or dumbbell-like joints, the distance between two adjacent ones is rather uniform, ranging from 100 to 150 nm. The bowl-like and dumbbell-like joints indicate batch growth behavior of the nanostructures [30]. The HRTEM in Figure 5d,e shows the features of the inner and outer walls in the hollow structures. The inner walls are essentially composed of chain-like aligned nanowires, while the outer walls are tubular sheaths, which are quite similar to an earlier literature report [20]. The outer walls composed of several layers can be distinctively observed in this study as shown in Figure 5d,f. The HRTEM also indicates the amorphous features of the nanowires confirmed by the SAED in the inset in Figure 5d. A typical EDX analysis confirms that the nanostructure is composed of Si and O with an O/Si atomic ratio of 1.8 as shown in Figure 5g, while the catalyst tips are Cu-rich particles as shown in the EDX analysis of Figure 5h, where the Ni signals are generated from the nickel grid supporting the nanostructures. The performance of biomedical devices involved with tubelike silica would be improved with more controllable features when the silica nanostructures with different tubelike morphologies could be applied in a controllable way [9,10].

In general, Si vapor source is mainly from the active oxidation of the silicon substrate in the presence of copper at high temperature, and O vapor source might come from the leakage of the vacuum system [26]. The copper layer breaks to small droplets at the heating process, then Cu-Si eutectic catalysts can be formed to initiate the nanostructure growth on the 3D carbon posts above the eutectic temperature, which matches with the photoresist carbonization process [26]. By dissolving Si and O source onto the catalyst droplet continuously, the silica nanostructures grow mainly through VLS and VQS mechanisms. The catalyst droplets with smoothly curved surfaces confirm the VLS process, as shown in Figures 2, 4, and 5[33]. Combining the results of the SEM and TEM, it is concluded that the Si vapor source concentration gradient would lead to unique morphology changes of nanostructures. The underlying reason could be due to the variations of reactive source concentration gradient inside different catalyst droplets caused by the local source concentration gradient in the environment, which is a key factor to determine the nanowire growth behavior and could result in diverse morphologies [32,34]. Furthermore, at a relatively lower temperature of 950°C, the active smaller catalysts in each catalyst might be concentric to initiate tubelike structures due to thermodynamic imbalance, which could be confirmed by Figure 4f [32].

It is also noticed that silica nanostructures are hard to observe at an annealing temperature below 900°C, while abundant silica nanowires are found all over the substrate when the temperature is above 1,050°C, which reminds us that temperature is another critical parameter to determine the growth behavior of silica nanostructures.

## Conclusions

In summary, the morphology evolution of silica nanostructures is induced by local Si vapor source concentration gradient as grown on a photoresist-derived 3D carbon microelectrode array at both 950°C and 1,000°C. Different silica nanostructure morphologies and their assemblies such as bamboo-, tube-, wire-, feature-, string-, and rope-like were obtained. Nanostructure branching, connecting, and batch growth phenomenon were also observed, which involved both VLS and VQS growth mechanisms. The study would help further understand the process of the nanowire formation and offers a potential method to design and synthesize nanostructures with controlled features. Meanwhile, it promotes an effective and controllable route to grow and integrate nanostructures on microstructures for applications in diverse fields.

## Competing interests

The authors declare that they have no competing interests.

## Authors’ contributions

DL and ZT designed the experiment. DL, SX, and SL performed the experiments. TS and XL contributed to material analysis. DL and ZT co-wrote the paper. WL is responsible for the correction of this paper. All authors read and approved the final manuscript.
